# Onopordopicrin from the new genus *Shangwua* as a novel thioredoxin reductase inhibitor to induce oxidative stress-mediated tumor cell apoptosis

**DOI:** 10.1080/14756366.2021.1899169

**Published:** 2021-03-18

**Authors:** Junmin Zhang, Zai-Qin Zheng, Qianhe Xu, Ya Li, Kun Gao, Jianguo Fang

**Affiliations:** School of Pharmacy, State Key Laboratory of Applied Organic Chemistry, and College of Chemistry and Chemical Engineering, Lanzhou University, Lanzhou, China

**Keywords:** Oxidative stress, thioredoxin, onopordopicrin, apoptosis, anticancer agent

## Abstract

Isolation and identification of natural products from plants is an essential approach for discovering drug candidates. Herein we report the characterization of three sesquiterpene lactones from a new genus *Shangwua*, e.g. onopordopicrin (ONP), C**2**, and C**3**, and evaluation of their pharmacological functions in interfering cellular redox signaling. Compared to C**2** and C**3**, ONP shows the most potency in killing cancer cells. Further experiments demonstrate that ONP robustly inhibits thioredoxin reductase (TrxR), which leads to perturbation of cellular redox homeostasis with the favor of oxidative stress. Knockdown of the TrxR sensitizes cells to the ONP treatment while overexpression of the enzyme reduces the potency of ONP, underpinning the correlation of TrxR inhibition to the cytotoxicity of ONP. The discovery of ONP expands the library of the natural TrxR inhibitors, and the disclosure of the action mechanism of ONP provides a foundation for the further development of ONP as an anticancer agent.

## Graphical Abstract

**Figure:**
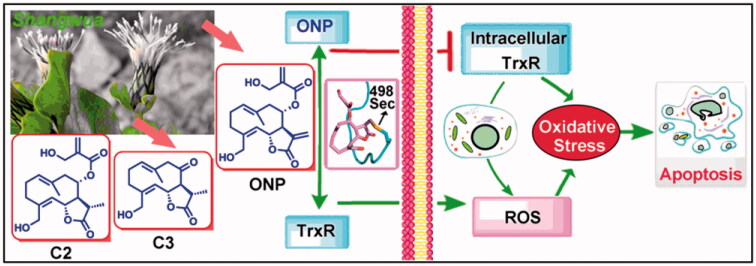

## 1. Introduction

Abundant scaffold diversity of natural products is almost beyond human imagination. Biological co-evolution endows natural products with perfect biocompatibility, resulting in natural products that can nearly match the three-dimensional configuration requirements of various protein targets in organisms^1^. Sesquiterpenoid derived from the medicinal plants *Asteraceae* has attracted wide attention from researchers due to targeting functional proteins to produce antitumor, antiviral, antibacterial, and antiinflammatory pharmacological activities^2^. *Shangwua*, a new genus of the *Asteraceae* family discovered in recent years, has been identified with three species, *e.g.* *Shangwua denticulata* (DC.) Raab-Straube & Yu J. Wang, *Shangwua jacea* (Klotzsch) Yu J. Wang & Raab-Straube, and *Shangwua masarica* (Lipsky) Yu J. Wang & Raab-Straube^3^. However, the medicinal value and secondary metabolite components for *Shangwua* have not been reported yet.

**Figure 1.**
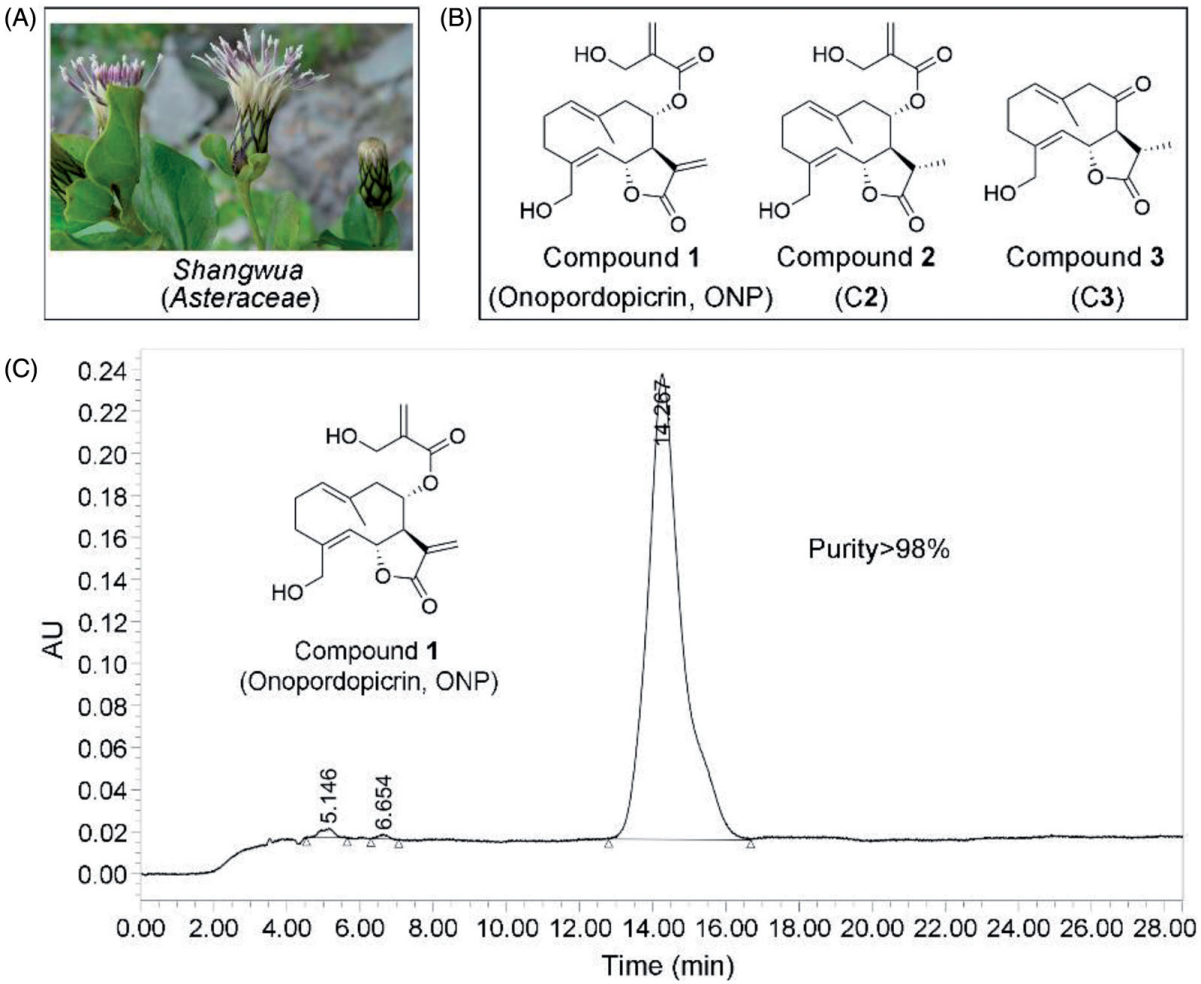
Compounds **1**-**3** isolated for the first time from *Shangwua*, a new genus of *Asteraceae*. (A) Tibetan plateau and Himalayas plant *Shangwua*. (B) Structure of compounds **1**–**3**, namely ONP, C**2,** and C**3**. (C) Purity analysis of ONP was analyzed by HPLC.

We thus isolated for the first time three sesquiterpene lactones from the whole plant of *Shangwua denticulate* (Figure 1(A)) collected from Ji-Long County, Tibet. Compounds **1**-**3** (Figure 1(B)) bearing the same lactone ring are onopordopicrin (Compound **1**, ONP), 11β,13-dihydro-19-desoxycnicin (Compound **2**, C**2**), and 8-oxo-15-hydroxygermacra-1(10)E, 4Z-dien-11βH-12, 6α-olide (Compound **3**, C**3**), respectively. ONP was previously reported to harbor antibacterial and antifungal^4^^,^^5^, antiplasmodial^6^, phytotoxic^7^, anti-ulcerogenic^8^, and anti-inflammatory activities^9^, while the biological activities of C**2** and C**3** are rarely reported. However, despite ONP exhibits cytotoxicity, whether ONP is effective for inhibiting tumor growth and acting mechanism remains unclear.

Alteration of redox homeostasis is a hallmark of cancer cells^10^. Thioredoxin reductase (TrxR) enzymes as a critical player in regulating cellular redox signaling are increasingly recognized as attractive targets for anticancer drugs^11–16^. Structurally, TrxR is a selenium-dependent enzyme with unique but indispensable selenocysteine (Sec) residue at the penultimate C-terminus^17^^,^^18^, thereby providing a preferential and selective target for covalent natural molecules^14^^,^^19^. Functionally, in addition to the key role of TrxR in redox regulation^20^^,^^21^, an ever-widening range of evidence has supported that TrxR highly enriched in many tumor types is essentially implicated with multiple steps of tumorigenesis and development^20–23^, and is a potential and promising target for the current anticancer drugs discovery^24–27^. As our continuing efforts in discovering therapeutic molecules derived from natural products that effectively interfere with cellular redox signaling^28–39^, we assessed herein the antitumor activity of ONP, C**2** and C**3**, and systematically reported a new mechanism by which ONP targets intracellular TrxR inhibition to cause oxidative stress-mediated tumor cell apoptosis. Mechanistically, the β-carbocation of the lactone ring in ONP covalently bound to the redox-active Sec residue at the C-terminus of TrxR, thereby leading to a decrease in TrxR activity. Importantly, carcinoma cells bearing high levels of oxidative stress and overexpressing TrxR under pathological conditions were selectively killed by ONP, showing a perfect antitumor effect. Moreover, the silence of TrxR expression by RNA interference enhanced the observed cytotoxicity of ONP while overexpression of the enzyme reduced the potency of ONP, thereby supporting that TrxR is essentially involved in the cellular effects of ONP. A novel TrxR inhibitor for the first time was isolated from *Shangwua* and its antitumor activity and action mechanism were reported, which not only revealed the new pharmacological activity of ONP but also provided insights for the development of the medicinal value of the new genus *Shangwua*.

## 2. Materials and methods

### 2.1. Chemistry

#### 2.2.1. Plant materials

The whole plant of *Shangwua* was collected from Ji-Long County, Tibet, China, in August 2016, and identified by Prof. Wang Yu-Jin of Lanzhou University. A voucher specimen (No. 20170728) was stored at the natural product laboratory of State Key Laboratory of Applied Organic Chemistry, Lanzhou University.

#### 2.2.2. General experimental procedures

200–300 mesh silica gel and 10–40 µm GF 254 silica gel plates (Qingdao Marine Chemical Factory, China), 150–200 mesh RP-C18 silica gel (Merck), and Sephadex LH-20 (Amersham Pharmacia Biotech UK Ltd.) were employed for column chromatography. Semipreparative HPLC with a reversed-phase C18 (150 × 10 mm, 10 µm) column was carried out on the isolation and purification of samples. ^1^H and ^13^C NMR spectroscopic data were used to a Varian Mercury-600BB or Bruker Avance III-400 instrument.

#### 2.2.3. Purity analysis

The purity analysis of ONP was performed on Waters pump 1525 and PDA 2998 series HPLC systems with reversed-phase C18 (4.6 × 150 mm, 5 µm) chromatographic columns at room temperature. ONP was dissolved in chromatographic methanol, and the injection volume was 10 µL. The mobile phases are methanol and water, and the flow rate is 1.0 mL/min. The maximum absorbance in the range of 210–400 nm is used as the detection wavelength.

#### 2.2.4. Chemical characterization

The chemical characterization spectrum and HPLC purity analysis diagram of compounds **1**-**3** were shown in the Supporting Information (Figues S1–S7). The isolated compounds **1**-**3** were employed in all subsequent activity experiments.

Compound **1** (ONP): yellow oil; ^1^H NMR (300 MHz, CD_3_OD, in ppm, *J* in Hz): 6.29 (1H, d, *J* = 1.2 Hz, H-4′), 6.10 (1H, br s, H-13), 5.97 (1H, br s, H-4′), 5.81 (1H, d, *J* = 2.8 Hz, H-13), 5.21 (1H, m, H-6), 5.13 (1H, m, H-8), 5.07 (1H, m, H-1) 4.95 (1H, d, J = Hz, H-5), 4.27 (1H, s, H-3′), 4.25 (1H, m, H-15a) , 4.01 (1H, d, *J* = 13.2 Hz, H-15b), 3.28 (1H, m, H-7), 2.62 (1H, m, H-3a), 2.55 (1H, m, H-9a) 2.33 (1H, m, H-2a), 2.20 (1H, m, H-2b), 2.01 (1H, m, H-3b), 1.54 (3H, s, H-14); ^13^C NMR: (75 MHz, CD_3_OD) 172.1 (C-12), 166.4 (C-1′), 145.6 (C-4), 141.9 (C-2′), 137.5 (C-11), 130.9 (C-1), 129.7 (C-5), 125.8 (C-4′), 125.3 (C-13), 78.6 (C-6), 74.4 (C-8), 61.6 (C-3′), 60.8 (C-15), 54.0 (C-7), 35.2 (C-3), 26.9 (C-2), 17.1 (C-14).

Compound **2** (C**2**): yellow oil; ^1^H NMR (300 MHz, CD_3_OD, in ppm, *J* in Hz): 6.26 (1H, s, H-4′a), 6.19 (1H, s, H-4′b), 5.25 (1H, m, H-8), 5.09 (1H, m, H-6), 5.04 (1H, m, H-1), 4.86 (1H, m, H-5), 4.30 (1H, s, H-3′), 4.26 (1H, d, *J* = 13.6 Hz, H-15a), 3.99 (1H, d, *J* = 13.6 Hz, H-15b), 2.69 (1H, m, H-11), 2.61 (1H, m, H-3a), 2.48 (1H, m, H-9), 2.38 (1H, m, H-7), 2.17 (1H, m, H-2), 2.01 (1H, m, H -2) -3a), 1.52 (1H, s, H-14), 1.36 (1H, d, *J* = 6.8 Hz, H-13); ^13^C NMR (75 MHz, CD_3_OD): 181.0 (C-12), 166.5(C-1′), 144.4(C-4), 142.0(C-2′), 133.7(C-10), 130.7(C-1), 130.3(C-5), 125.5(C-4′), 77.9 (C-6), 74.8 (C-8), 61.7 (C-3′), 60.6 (C-15), 59.1 (C -7), 49.9 (C-9), 35.2 (C- 11), 30.7 (C-3), 26.7 (C-2), 17.3 (C-13), 16.9 (C-14).

Compound **3** (C**3**): yellow oil; ^1^H NMR (300 MHz, CD_3_OD, ppm, *J* in Hz): 5.19 (1H, m, H-1), 5.02 (1H, d, *J* = 10.2 Hz, H-5), 4.93 (1H, m, H-6), 4.10 (1H, d, *J* = 13.8 Hz, H-15a), 3.78 (1H, d, *J* = 13.8 Hz, H-15 b), 3.32 (1H, d, *J* = 9.9 Hz, H-15a), 3.11 (1H, m, H-7), 2.95 (1H, d, *J* = 9.6 Hz, H-15b), 2.59 (1H, m, H-3a), 2.35 (2H, m, H-2), 2.06 (1H, m, H-3b), 1.42 (3H, s, H-14), 1.18 (3H, d, *J* = 6.6 Hz, H-15a); ^13^C NMR (75 MHz, CD_3_OD) 204.0 (C-8), 177.7 (C-12), 145.7 (C-4), 132.5 (C-1), 127.8 (C-5), 126.5 (C-10), 75.6 (C-6), 64.3 (C-7), 59.6 (C-15), 57.5 (C-9), 40.6 (C-11), 33.4 (C-3), 24.8 (C-2), 16.0 (C-14), 13.6 (C-13).

### 2.2. Enzymes and materials

The recombinant rat TrxR1 was a gift from Prof. Arne Holmgren (Karolinska Institute, Sweden). The recombinant U498C TrxR1 (Sec→Cys) mutant and the *Escherichia coli* (*E. coli*) Trx were prepared by our Lab as described^36^. Both the plasmids: shTrxR1, shRNA specifically targeting *TrxR1*, shNT, non-targeting control *shRNA*, and the cell lines: HEK-*TrxR1* cells, HEK cells stably overexpressing *TrxR1*, HEK-*IRES* cells, HEK cells stably transfected with a vector were gifts from Prof. Constantinos Koumenis (University of Pennsylvania, USA)^40^^,^^41^. The preparation of HeLa-sh*NT* and HeLa-sh*TrxR1* cell lines were described^37^.

Dimethyl sulfoxide (DMSO), Dulbecco′s modified Eagle’s medium (DMEM), G418, puromycin, 2′, 7′-dichlorfluorescein diacetate (DCFH-DA), Hoechst 33342, dihydroethidium (DHE), insulin (from bovine), DL-dithiothreitol (DTT), N-acetyl-Asp-Glu-Val-Asp-p-nitroanilide (Ac-DEVD-pNA), and 3-[(3-Cholamidopropyl) dimethylammonio]-1-propanesulfonate (CHAPS) were obtained from Sigma-Aldrich (St. Louis, USA). Penicillin, MTT and streptomycin were products of Amresco (Solon, OH). NADPH was purchased from Roche (Mannheim, Germany). Fetal bovine serum (FBS) was a product of Sijiqing (Hangzhou, China). DTNB (5,5′-dithiobis-2-nitrobenzoic acid) was a product of J&K Scientific (Beijing, China). Bovine serum albumin (BSA), Trypan blue, phenylmethylsulfonyl fluoride (PMSF), and Na_3_VO_4_ were products of Beyotime (Nantong, China). The propidium iodide (PI) and Annexin V-FITC apoptosis assay kit was a product of Zoman Biotech (Beijing, China). HPLC grade methanol and acetonitrile were products of Merck (Darmstadt, Germany). All other chemicals used were of analytic grade.

### 2.3. Cell cultures

Different cell lines (HeLa, HepG2, A549, L02, and BEAS-2B) were obtained from the Shanghai Institute of Biochemistry and Cell Biology, Chinese Academy of Sciences, and were cultured under a standard culture condition (5% CO_2_ atmosphere, 37 °C incubator with a humidified; DMEM supplemented with 10% FBS, 2 mM glutamine, and100 units·mL^−1^ streptomycin/penicillin). HEK-*TrxR1* and HEK-*IRES* cells were grown in the standard culture conditions supplemented with an additional 0.1 µM sodium selenite and 0.4 mg·mL^−1^ G418. HeLa-sh*TrxR1* and HeLa-sh*NT* cells were cultured under the standard culture conditions supplemented with an additional 1 µg·mL^−1^ puromycin.

### 2.4. Cell viability analysis

#### 2.4.1. MTT assay

Cells (5 × 10^3^−1 × 10^4^/well) from above different cell lines were incubated with agents and grown in triplicate in a 96-well plate for 24, 48, or 72 h at 37 °C in a final volume of 100 µL. The cells treated with 0.1% DMSO alone were the control group. After incubated for the designed time, MTT reagent (5 µL/well, 5 mg·mL^−1^) was added and continued culturing for an additional 4 h at 37 °C. Formazan crystals then were solubilized in solvent 100 µL extraction buffer (10% SDS, 0.1% HCl, and 5% iso-butanol). The absorbance was read at 570 nm using a microplate reader (Thermo Scientific Multiskan GO, Finland)) to calculate the cell viability.

#### 2.4.2. Trypan blue exclusion assay

Cells (2 × 10^5^/well) were seeded in a 12-well plate and were cultured overnight. The cells were further treated with indicated concentrations of ONP (20 and 40 µM) for 24 h. The control cells were treated with 0.1% DMSO alone. Then, the cells were stained with trypan blue (0.4%, w/v), and the number of dead (stained) cells and viable (non-stained) cells were counted.

### 2.5. Apoptosis assays

#### 2.5.1. Hoechst 33342 staining

Hoechst 33342 dye is used to detect the morphological changes of apoptotic cells. Briefly, HeLa cells (2 × 10^5^) were seeded in a 12-well plate and were cultured overnight. The cells subsequently were incubated with a designed concentration of ONP for 24 h. Hoechst 33342 dye (5 µg·mL^−1^) was added and continued culturing for 30 minutes. Rinsed HeLa cells with the medium to remove the remaining Hoechst 33342. The stained nuclei were photographed under a fluorescent microscope.

#### 2.5.2. Annexin V-FITC/PI staining

HeLa cells (5 × 10^5^) were plated in a 6-well plate and were cultured overnight. The cells were then incubated with different concentrations of ONP for 24 h. Subsequently, the cells were harvested and washed with PBS. According to the Annexin V-FITC/PI double staining apoptosis detection kit (Zoman Biotech, Beijing, China), the apoptotic cells were determined and analyzed by flow cytometry with Cell Quest software (BD Biosciences, USA).

#### 2.5.3. Caspase 3 activity assay

HeLa cells were treated with indicated concentrations of ONP for 24 h. The cells were then collected and lysed with RIPA buffer. Total cellular protein contents were quantified by the Bradford procedure. The extracts containing 60 µg of total proteins were incubated with the protease activity assay mixture (0.2 mM Ac-DEVD-pNA, 0.1% CHAPS, 5% glycerol, 2 mM EDTA and 10 mM DTT in 50 mM Hepes, pH 7.5) at 37 °C for 2 h in a final volume of 100 µL. The caspase 3 activity was determined by measuring the absorbance at 405 nm.

### 2.6. Measurement of intracellular ROS

DCFH-DA or DHE probes were employed to assess ROS level. HeLa cells (2 × 10^5^) were plated into a 12-well plate and allowed to adhere. HeLa cells were incubated with 0, 20, or 40 µM ONP for 5 h, then removed the medium and subsequently treated with DCFH-DA (10 µM) or DHE (10 µM) in fresh FBS-free medium for 30 min at 37 °C in dark. The images were acquired on a fluorescent microscope (Floid Cell Imaging Station, Thermo Fisher).

### 2.7. Assay of cellular TrxR activity

#### 2.7.1. TRFS-green-based live-cell imaging TrxR activity assay

TRFS-green is a specific TrxR probe by our previously established^42^. HeLa cells (2 × 10^5^) were plated into a 12-well plate and allowed to adhere. The HeLa cells were incubated with 0, 10, 20, or 40 µM of ONP or 40 µM C**2** or C**3** for 8 h. Then the cells were removed from the medium and subsequently treated with TRFS-green (10 µM) in a fresh FBS-free medium for 4 h at 37 °C in dark. The cells were washed to remove the residual TRFS-green. The cells were imaged under a fluorescent microscope (Floid Cell Imaging Station, Thermo Fisher).

#### 2.7.2. Fast-TRFS-based cell lysate TrxR activity assay

Fast-TRFS-based cell lysate assay is the rapid detection of TrxR activity in crude protein as the source of TrxR by our previously described method^43^. Briefly, HeLa cells were plated in 100-mm culture dishes and were cultured overnight. The cells then were collected and lysed with RIPA buffer. Total cellular protein contents as the source of TrxR were quantified by the Bradford procedure. Subsequently, the HeLa cell lysate (0.3 mg·mL^−1^) was incubated with NADPH (100 µM) for 5 min at 37 °C. Test drug, ONP (10, 20, or 40 µM each), and blank sample (0.1%DMSO) were then added and the mixture was continued to incubate for 1 h. The probe Fast-TRFS (10 µM) and NADPH (100 µM) were added to initiate the enzymatic reduction of Fast-TRFS. The fluorescence change at 460 nm was recorded (*λ*ex = 345 nm) for 10 min on a fluorescent plate reader (Tecan Infinite M200), and the rate of fluorescence increase within the initial 5 min was calculated. The relative TrxR activity was expressed as the percentage of the DMSO-treated sample.

#### 2.7.3. Trx-mediated endpoint insulin reduction assay

HeLa cells were treated with different concentrations of ONP, C**2**, and C**3** for 12 or 24 h. The cells were washed and harvested. Subsequently, the total cellular proteins were extracted and quantified by the RIPA buffer and the Bradford procedure, respectively. The intracellular TrxR activity was measured by the endpoint insulin reduction assay described as our published procedures^30^^,^^37^.

### 2.8. Pure TrxR activity assays

The NADPH-reduced TrxR (170 nM) or U498C TrxR (700 nM) was incubated with 0, 2.5, 5, 10, and 20 µM of ONP or C**3** in a 96-well plate at room temperature for 0.5, 1, or 2 h, and the final incubation volume of the mixture was 50 µL. Then added 50 µL TE buffer (50 mM Tris-HCl, 1 mM EDTA, pH 7.5) containing 2 mM DTNB and 200 µM NADPH, recorded the linear increase during the initial 3 min in absorbance at 412 nm^30^^,^^37^. The activity was expressed as the percentage of the control and the same amounts of DMSO were added to the control experiments.

### 2.9. Molecular docking simulation

The crystal structure of rat TrxR1 (PDB code 3EAN, chain A) was employed in the present docking study as we described previously^29^. The residue Sec498 in chain A was selected and further prepared in the protein preparation wizard module as the reactive residue involved in the Michael addition. In addition, the residue Sec498 was also set as the centroid of the docking pocket. The docking simulation was carried out with the default parameters.

### 2.10. Statistics

All experiment data are presented as the mean ± S.E. Statistical differences between two groups were assessed by Student’s t-test. *p* < 0.05 was used as the criterion for statistical significance.

## 3. Results and discussion

### 3.1. Chemical isolation and characterization

The dried whole plant (2.375 kg) of *Shangwua* (Figure 1(A)) was pulverized and extracted with 95% ethanol (3 × 5 L) at room temperature. The mixture was later filtered and concentrated under reduced pressure to provide a crude extract (152.8 g). Subsequently, the crude extract was extracted with ethyl acetate, and the resulting extract (98.9 g) was eluted with ethanol/water (30, 50, 80, 90 and 100%) through macroporous adsorption resin. The 80% ethanol eluted fraction was subjected to silica gel column chromatography and eluted with petroleum ether/acetone to obtain six sub-fractions (Fr. A-F). The Fr. B subfraction was eluted through a reversed-phase silica gel column with a gradient of methanol: water (3:7–0:1). The Fr.B3 part obtained above was separated by Sephadex LH-20 and semi-preparative high-performance liquid chromatography (HPLC) to finally obtain the pure compound **1** (20 mg). Likewise, Fr. E subfraction was directly separated by reversed-phase silica gel column and semi-preparative HPLC (methanol: water = 3:2) to obtain compound **2** (6 mg). Subsequently, the Fr. D subcomponent was eluted by normal phase silica gel (petroleum ether: acetone = 3:1-1:1) to obtain Fr. D2. Then, Fr. D2. was separated and purified by Sephadex LH-20 and semi-preparative HPLC to obtain compound **3** (10 mg).

The structures of compounds **1**–**3** were characterized by ^1^H and ^13^C NMR, and were determined to be onopordopicrin (ONP, Figure 1(B))^4^, 11β,13-dihydro-19-desoxycnicin (C**2**, Figure 1(B))^44^, and 8-oxo-15-hydroxygermacra-1(10)E, 4Z-dien-11βH-12, 6α-olide (C**3**, Figure 1(B))^45^, respectively. The purity of ONP is quantified by HPLC (purity >98%, Figure 1(C)).

### 3.2. Inhibiting tumor cell growth

**Figure 2.**
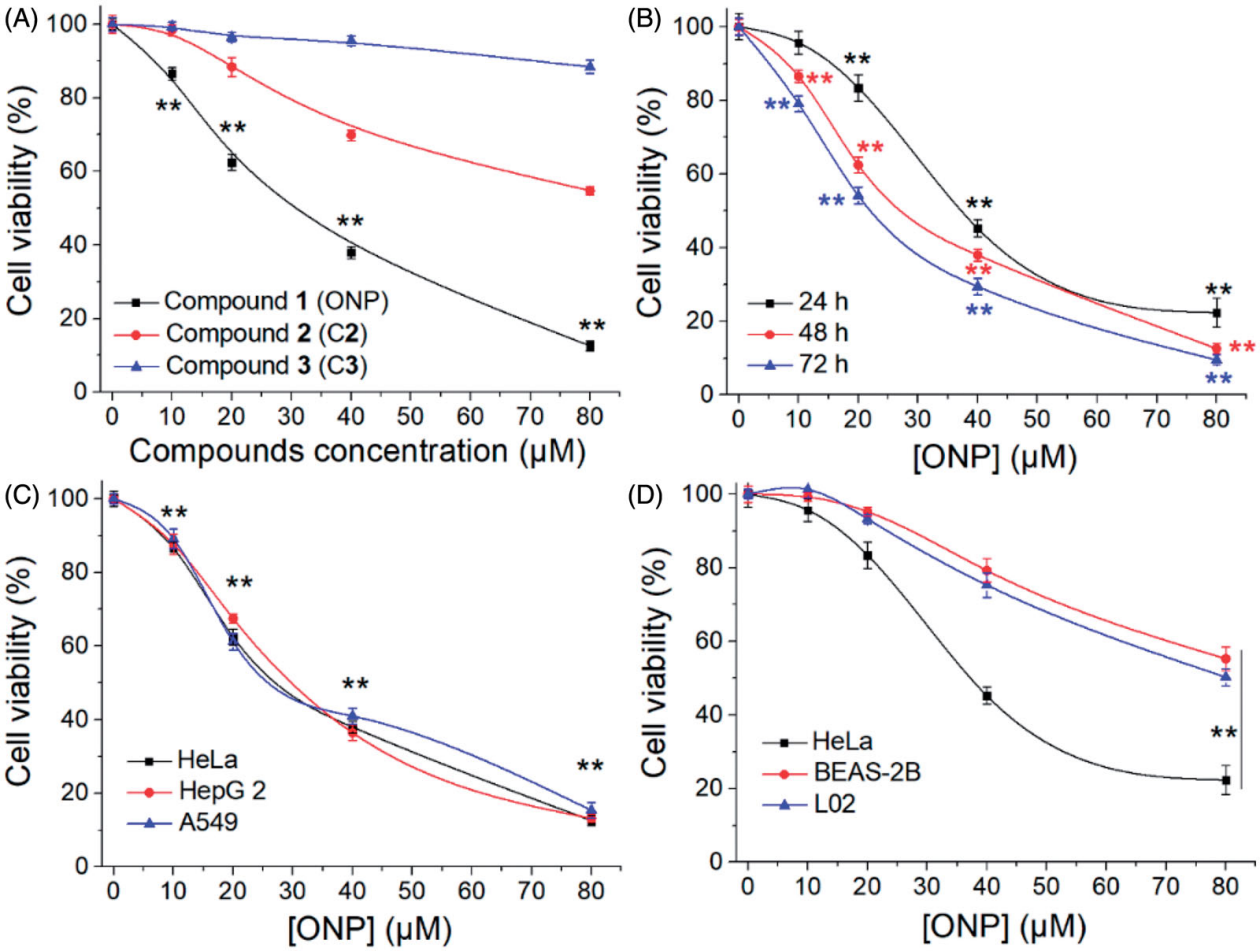
Inhibiting tumor cell growth by ONP. (A) Cytotoxicity of ONP, C**2,** and C**3** to HeLa cells. After treating HeLa cells with the indicated concentrations of ONP, C**2,** and C**3** for 48 h, the cell viability of HeLa cells was determined by the MTT method. (B) Inhibition of the growth of HeLa cells in a time-dependent manner by ONP. HeLa cells were treated with ONP at designated concentrations for 24, 48, and 72 h, and the cell survival rate was detected by the MTT method. (C) Cytotoxicity of ONP towards multiple tumor cell lines. After different concentrations of ONP were incubated with HeLa, HepG2, and A549 cells for 48 h, the cell viability was detected by the MTT method. (D) Selectivity of ONP for tumor cells. The designated concentrations of ONP acted on HeLa cells, BEAS-2B, and L02 cells for 24 h, and the cell viability was detected by the MTT method. After treatment of HeLa cells, BEAS-2B, and L02 cells with ONP at specified concentrations for 24 h, the cell viability was detected by the MTT method. Data are expressed as mean ± S. E. of three experiments. **p < 0.01 versus the control groups.

Given that the lack of research on *Shangwua* and its secondary metabolites, we urgently tested the cytotoxicity of the preceding three isolated compounds on tumor cells. As shown in Figure 2(A), the result showed that ONP can significantly inhibit the growth of human cervical cancer HeLa cell line with an IC_50_ value of 20 µM at 48 h. However, C**2** has little cytotoxicity and C**3** is almost non-cytotoxic under our experimental conditions towards HeLa cells (Figure 2(A)). In addition, the cytotoxicity of ONP towards HeLa cells also exhibited a time dependence (Figure 2(B)). To further confirm the cytotoxicity of ONP to tumor cells, we selected human liver cancer cell line HepG 2 and human lung cancer cell line A549, together with HeLa cells, to assess the growth inhibitory effect of ONP on the tumor cells. Satisfactory results presented that ONP had a remarkable activity of repressing the growth of tumor cells on the above tumor cell lines after 48 h incubation (Figure 2(C)). More noteworthy is that ONP could selectively kill tumor cells but was less cytotoxicity to normal cells such as human lung epithelial cells BEAS-2B and human liver epithelial cells L02 under the same experimental conditions (Figure 2(D)). These results indicated that ONP, C**2** and C**3** isolated from *Shangwua* have radically different cytotoxicity to HeLa cells due to diversities in their structural fragments. Moreover, ONP with a unique cytotoxic selectivity established by these data deserves a further investigation of its action mechanism for ablating tumor cells.

### 3.3. Inducing tumor cell apoptosis

**Figure 3.**
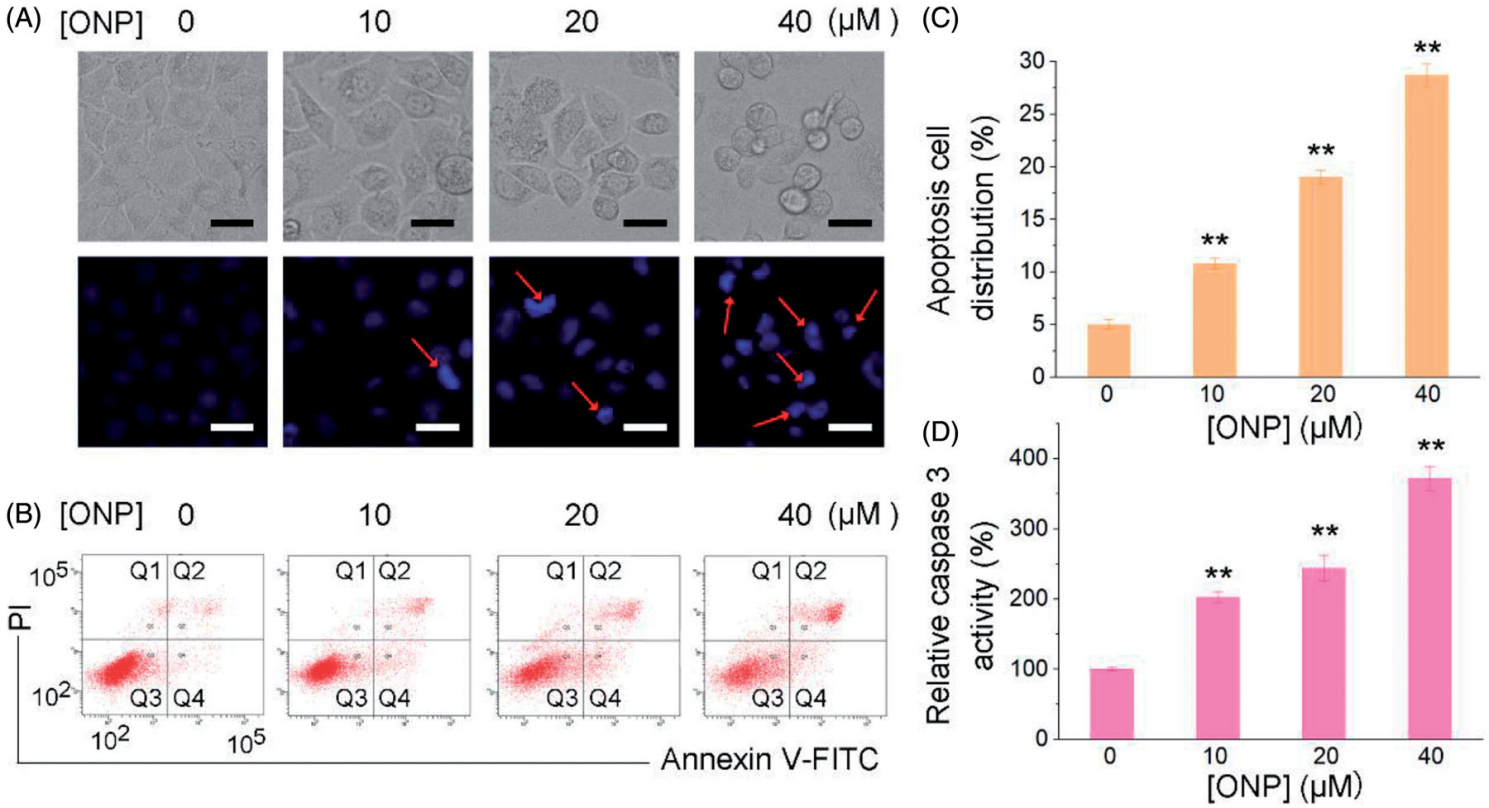
Killing HeLa cells by ONP primarily *via* inducing apoptosis. (A) Nuclear morphological changes after ONP treatment. HeLa cells were incubated for 24 h with 0, 10, 20, or 40 μM of ONP treatment, and the nuclei were stained by Hoechst 33342. Nuclear morphology changes then were observed under a fluorescence microscope. The bright filed pictures (top panel) and the fluorescence pictures (bottom panel) were imaged and acquired. Scale bars: 20 μm. Arrows refer to the condensed and irregular nuclei with bright fluorescence stained by Hoechst 33342 in (A). (B) Assessment of apoptosis by Annexin V-FITC/PI double staining assay. HeLa cells were treated with 0, 10, 20, or 40 μM of ONP treatment for 24 h, and the numbers of apoptotic cells were measured by the Annexin V-FITC/PI double staining assay. (C) Quantification of apoptotic cells (Q2 and Q4) from (B) by flow cytometry. (D) Activation of caspase 3 in HeLa cells by ONP. HeLa cells were incubated with 0, 10, 20, or 40 μM of ONP treatment for 24 h, and the caspase 3 activity in the cellular extracts was measured by a colorimetric assay. Data are expressed as mean ± S. E. of three experiments. **p < 0.01 versus the control groups.

ONP with similar properties as the activities of sesquiterpene lactones reported so far can significantly suppress the growth of tumor cells. Functionally, most of the reported sesquiterpene lactones inhibit tumor cell proliferation *via* inducing tumor cell apoptosis^46–48^. Next, we thus sought to confirm whether ONP can induce tumor cell apoptosis. As shown in Figure 3, we had fully demonstrated that ONP can promote HeLa cell apoptosis through various methods. First, we stained with Hoechst 33342 to observe whether HeLa cells treated with diverse concentrations of ONP can undergo morphological changes of apoptosis. As shown in Figure 3(A), our experimental results displayed that after different concentrations of ONP acted on HeLa cells, especially treatment with 40 µM, ONP could significantly cause HeLa cell nuclei to shrink and shine, a typical morphological change of apoptosis. To further confirm the apoptotic morphological changes produced by ONP-treated HeLa cells to eventually induce apoptosis, Annexin V-FITC and propidium iodide (PI) double staining reagents were employed to stain again and detect the number of apoptotic cells by flow cytometry to evaluate the ability of ONP to induce HeLa cell apoptosis. The results in Figure 3(B) intuitively exhibited that ONP effectively induced HeLa cell apoptosis. The number of apoptotic cells increased with the enhancement of ONP treatment concentration (Figure 3(C)), indicating directly that ONP could induce HeLa cell apoptosis in a concentration-dependent manner. In addition, our results showed that ONP induced HeLa cell apoptosis in a caspase 3 activity-dependent manner. As shown in Figure 3(D), the activity of intracellular caspase 3 activity increased remarkably with the increase of ONP treatment concentration, which further confirmed that ONP kills cells basically in the form of apoptosis. Taken together, ONP can significantly induce caspase 3-dependent apoptosis in HeLa cells in a concentration-dependent manner to contribute to the ability to kill tumor cells.

### 3.4. Increasing ROS accumulation

**Figure 4.**
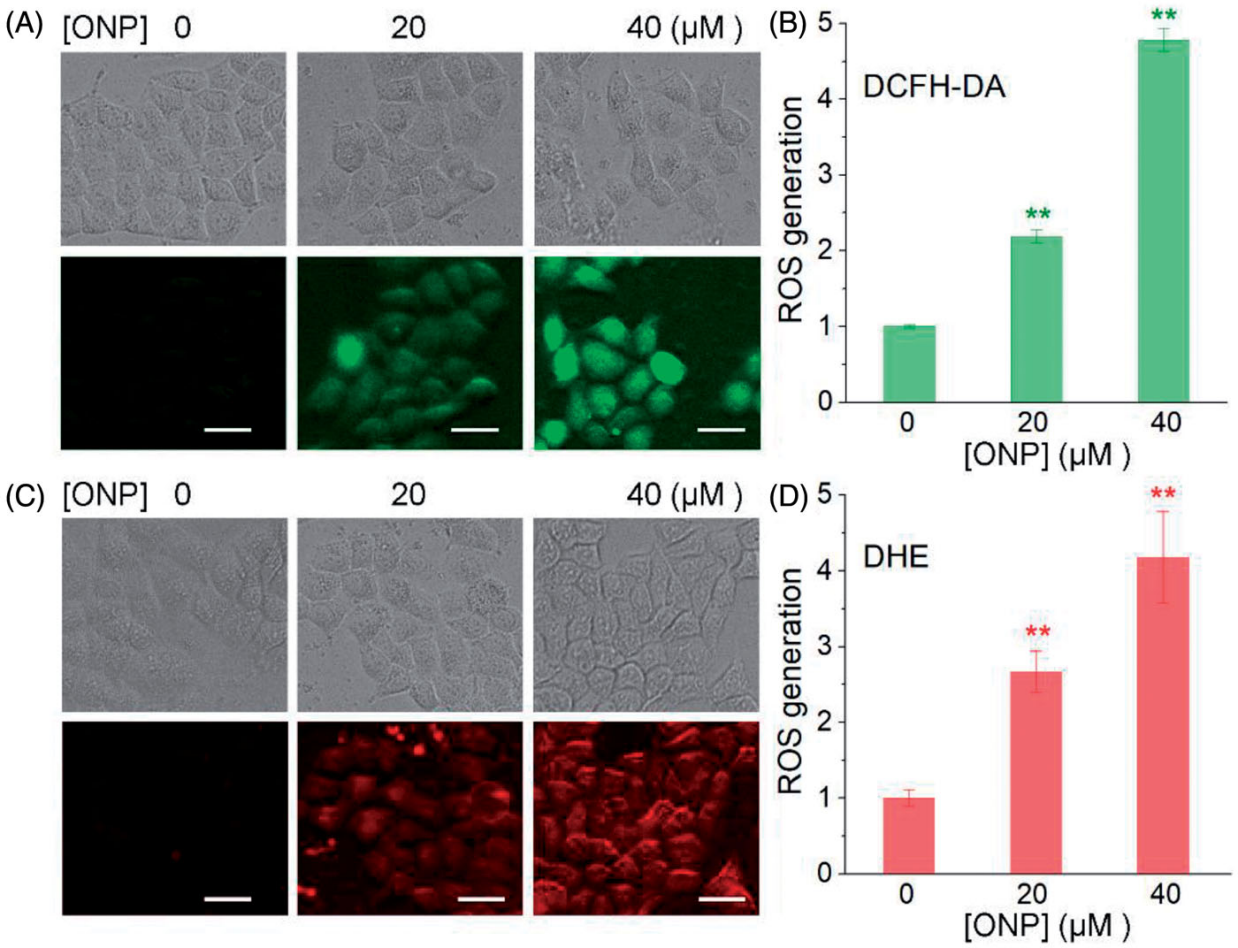
Induction of ROS accumulation in HeLa cells. Accumulation of ROS in HeLa cells by DCFH-DA staining (A) or DHE staining (C). HeLa cells were treated with 20 or 40 μM of ONP for 24 h, followed by incubation with the ROS probe DCFH-DA (10 μM) or superoxide probe DHE (10 μM) for 30 min. The bright-field (top panel) and the fluorescent-field (bottom panel) pictures from three independent experiments were imaged and acquired by an inverted fluorescence microscope. The fluorescence intensity in individual cells from (A) and (C) was quantified by ImageJ versus the control group and was shown in (B) and (D). **p < 0.01 versus the control groups. Scale bars: 20 μm.

We then explored the mechanism by which ONP induced HeLa apoptosis and showed selective inhibition of tumor cell growth. Considering that one of the biochemical characteristics of carcinoma cells is that they exhibit a higher level of oxidative stress than normal cells, and further stimulation by cytotoxic agents leads to preferentially induce tumor cell apoptosis^10^^,^^49^. We thus observed the changes in ROS levels in HeLa cells treated with ONP to validate the elevated oxidative stress level is the mechanism by which ONP selectively induces tumor cell apoptosis. We administered a fixed concentration of ONP to HeLa cells for different periods and alternately detected the level of intracellular ROS by DCFH-DA probe. Our results clearly showed that the intracellular ROS level reached the highest level after ONP treated HeLa cells for 5 h. As shown in Figure 4(A), the green fluorescence enhanced dramatically after treating HeLa cells with 20 and 40 µM ONP for 5 h, suggesting that a large amount of ROS was produced. The effect of 40 µM treatment was much better than that of 20 µM, indicating that excessive accumulation of ROS is also concentration-dependent (Figure 4(B)). To confirm this result, we used a DHE probe with a different fluorescence wavelength from DCFH-DA to detect again the level of ROS especially superoxide anion in ONP-treated HeLa cells. As shown in Figure 4(C,D), after treatment of HeLa cells with a concentration of 20 µM and 40 µM ONP, intracellular red fluorescence increased remarkably, which once more proved that excessive accumulation of ROS was generated in the HeLa cells treated with ONP. A cascade of ROS generation inevitably broke the upper limit of the cellular adaptive oxidative stress response, and eventually provoked HeLa cell apoptosis.

### 3.5. Inhibiting cellular TrxR activity

**Figure 5.**
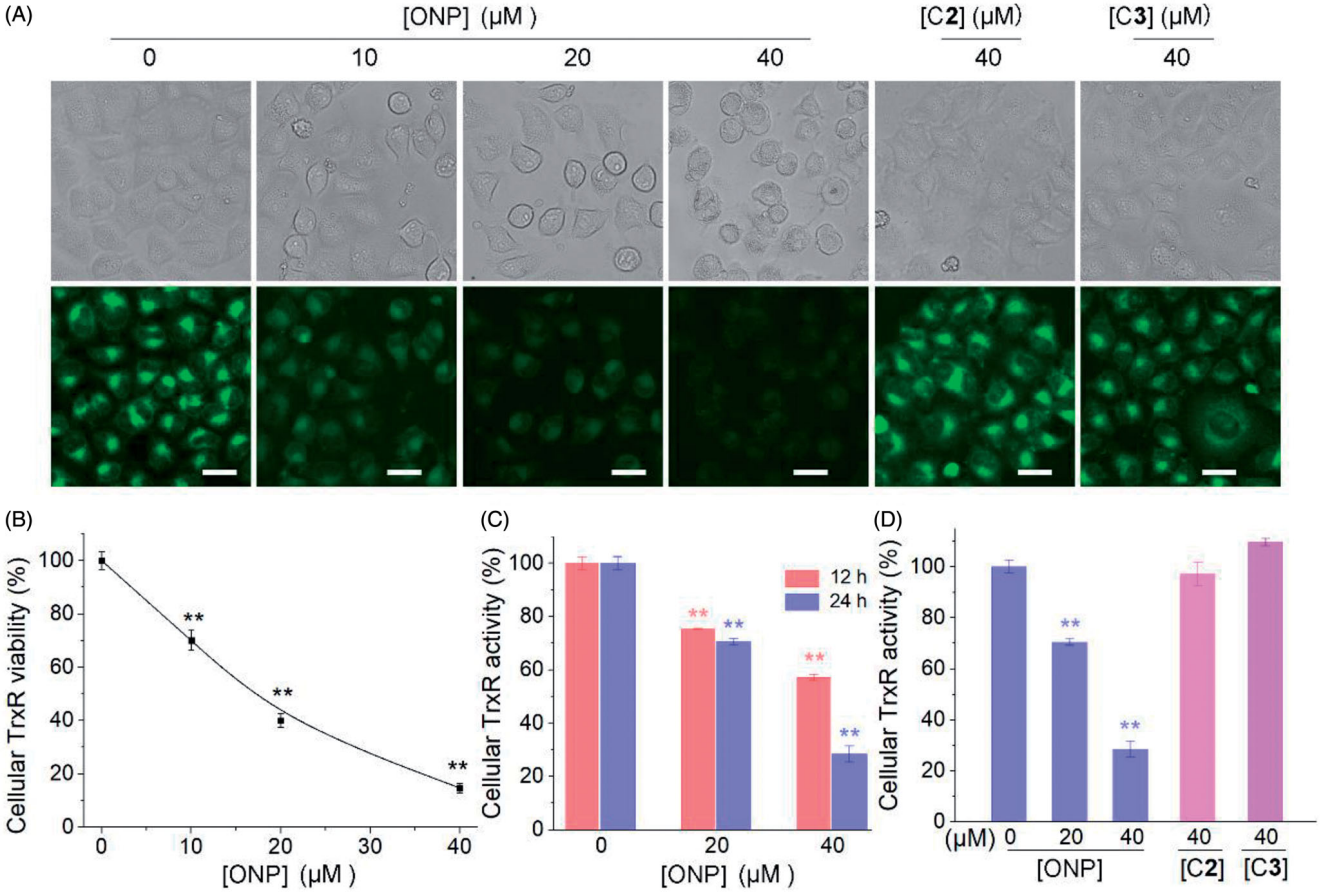
Inhibiting cellular TrxR activity by ONP. (A) TRFS-green-based live-cell imaging TrxR activity assay. HeLa cells were incubated with 0, 10, 20, or 40 μM of ONP or 40 μM C**2** or C**3** for 12 h. Cellular TrxR activity was assessed by TRFS-green. The fluorescence images were acquired by inverted fluorescence microscopy. (B) Fast-TRFS-based cell lysate TrxR activity assay. HeLa cell lysate (0.3 mg·mL^−1^) was incubated with NADPH (100 µM) for 5 min at 37 °C. ONP (0, 10, 20, or 40 µM) and blank sample (0.1% DMSO) were added to and the mixture was continued to incubate for 1 h. The probe Fast-TRFS (10 µM) and NADPH (100 µM) were added to initiate the enzymatic reduction of Fast-TRFS. The fluorescence change at 460 nm was recorded (λex = 345 nm) for 10 min on a fluorescent plate reader (Tecan Infinite M200), and the rate of fluorescence increase within the initial 5 min was calculated. The relative TrxR activity was expressed as the percentage of the DMSO-treated sample. Inhibition of intracellular TrxR activity by ONP in a concentration and time-dependent manner (C), and the difference of inhibition of cellular TrxR activity by ONP, C**2,** and C**3** (D) were assayed by the Trx-mediated endpoint insulin reduction. After the HeLa cells were treated with varying concentrations of ONP, C**2,** and C**3** for 12 or 24 h, the enzyme activity of TrxR in HeLa cells was determined by the Trx-mediated endpoint insulin reduction assay. Scale bars: 20 μm. Data are expressed as mean ± S. E. of three experiments. ** *p* < 0.01 versus the control group.

As one of the essential antioxidant enzymes in cells is TrxR. If the TrxR activity is inhibited, the intracellular antioxidant capacity for tumor cells is partially lessened, thereby causing the above-mentioned substantial accumulation of ROS^39^^,^^50^. We thus examined the effect of ONP on cellular TrxR activity. Firstly, the TRFS-green probe established by our group^42^ was invested to detect the effects of the ONP, C**1**, and C**2** on TrxR activity in HeLa cells. As shown in Figure 5(A), ONP harbored an effective ability to suppress intracellular TrxR activity concentration-dependently from the qualitative results of fluorescence microscopy imaging. While compounds C**2** and C**3** remained almost silent towards TrxR activity in cells at our observed concentrations (Figure 5(A)). Likewise, the fluorescence quantification results were also demonstrated by Fast-green, another detection probe for TrxR (Figure 5(B))^43^. To further validate the qualitative and quantitative results of the probes cell imaging, we employed the classic Trx-mediated insulin reduction method to assess the ability of ONP, C**2**, and C**3** to repress TrxR activity in HeLa cells (Figure 5(C)). Consistent with expectations, ONP remarkably suppressed intracellular TrxR activity in a concentration-and time-dependent manner (Figure 5(C)). Whereas there was no significant difference in the inhibitory effects of C**2** and C**3** on intracellular TrxR activity compared with the control group consistent with the probe test results (Figure 5(D)). By comparing the structures of ONP, C**2**, and C**3**, the molecular skeleton bearing α, β unsaturated ketone fragments seems to be highly crucial for their abilities to restrain intracellular TrxR activity, which requires further validation. Collectively, our data strongly confirmed that ONP can excellently inhibit TrxR activity in HeLa cells.

### 3.6. Confirmation of the interaction site

**Figure 6.**
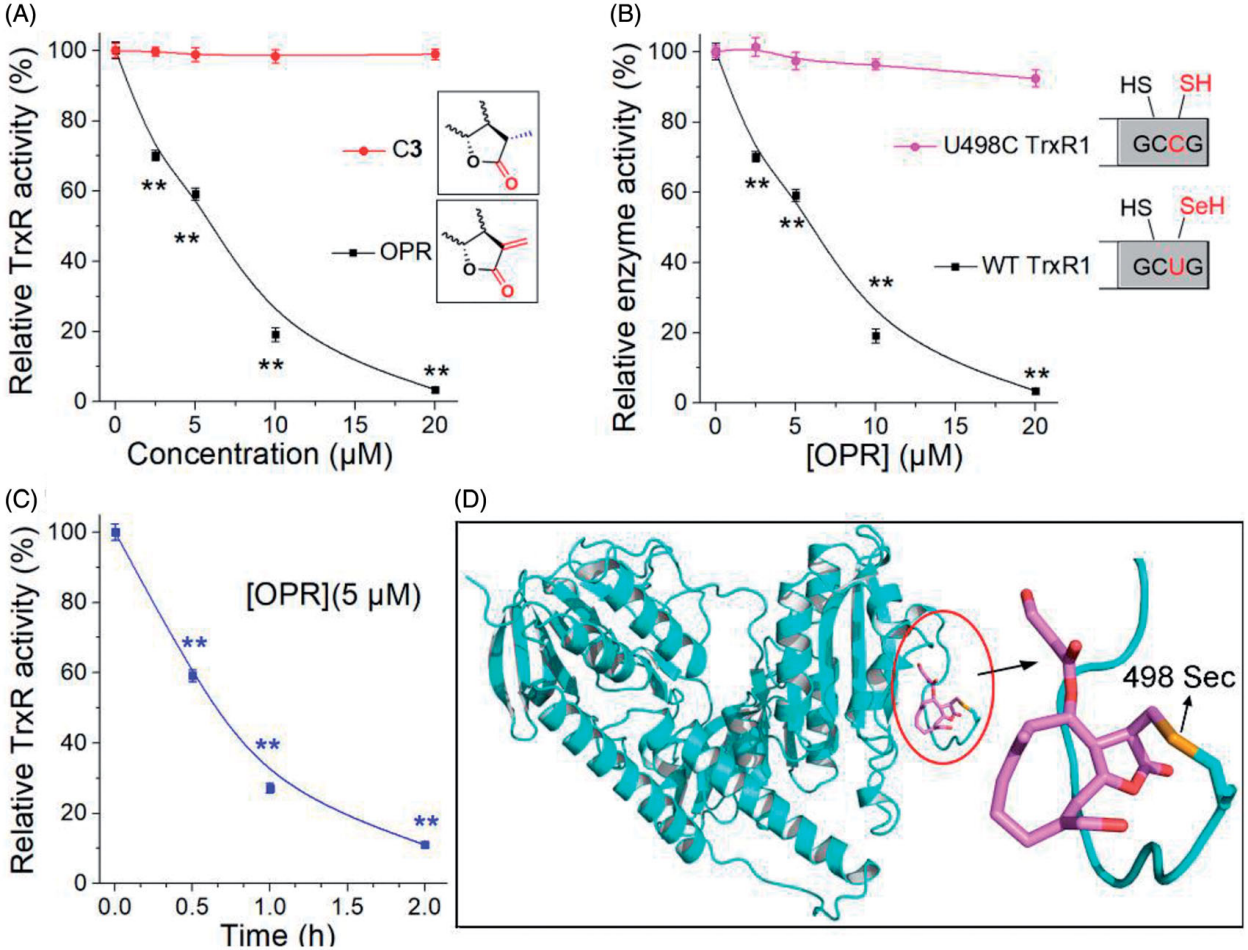
Evidence for the interaction site between ONP and TrxR. (A) ONP and C**3** selectively inhibited pure TrxR. (The illustration is the structural difference between ONP and C**3**). (B) Varying concentrations of ONP showed the difference in activity inhibition of WT TrxR and U498C TrxR1. (The illustration exhibited the difference between WT TrxR and U498C TrxR1). (C) ONP at a concentration of 5 μM inhibited the activity of pure TrxR in a time-dependent manner. The enzyme activities in (A), (B), and (C) are all detected by the DTNB method. The NADPH pre-reduced enzyme was incubated with different concentrations of ONP or C**3** for 1 h, and then the enzyme activity in the system was detected by the DTNB method. (D) Covalent docking for ONP with the C-terminal site 498 of the chain A of the mouse TrxR1. The docking experiment was conducted using the covalent docking protocol in the SchrödingerSuite 2015-1 program. The monomer of TrxR1 is represented by a cyan cartoon. The interacting residues in the ONP and active site are shown in orange and purple sticks. Data are expressed as mean ± S. E. of three experiments. **p < 0.01 versus the control groups.

To confirm the key performance of α, β-unsaturated ketone fragments on the lactone ring in the interaction between ONP and TrxR, we explored the interaction of ONP and C**3** with pure TrxR. The results were shown in Figure 6(A). Compared with C**3**, ONP could substantially inhibit the pure TrxR activity in a concentration-dependent manner. The vital structural difference between C**3** and ONP was that the α, β-unsaturated ketone fragment on the lactone ring (the inset of Figure 6(A)). ONP bearing this key fragment had remarkable enzyme inhibitory activity while C**3** was almost silenced, heavily validating the importance of α, β-unsaturated ketone fragment in weakening TrxR activity consistent with the literature^51^. In addition, ONP at a concentration of 5 µM also had the ability to inhibit pure TrxR in a time-dependent manner (Figure 6(C)), and the inhibition ability was nearly saturated after incubation for 1 h. Existing evidence highly confirms that the redox-active Sec residue at the C-terminus of TrxR plays a cardinal role in the inhibition of TrxR activity by small molecules^52^. The reason is that compared with the homologous cysteine (Cys), Sec has stronger nucleophilicity and stronger resistance to irreversible oxidation^53^. The high nucleophilicity of Sec residues and exposure to the outer surface of the enzyme render TrxR assailable to be modified by electrophiles^11^^,^^13^. To validate whether Sec residues also play a key role in the interaction between ONP and TrxR, we constructed a control protein for the WT TrxR1, which mutated the Sec at position 498 of the C-terminus to Cys to obtain the U498C TrxR1 mutant enzyme (the inset of Figure 6(B)). The effect of ONP on wild-type TrxR1 and recombinant U498C TrxR1 was shown in Figure 6(B). ONP hardly inhibited U498C TrxR1, suggesting that the site where ONP interacted with pure TrxR is Sec residue at position 498. We then docked the receptor rat TrxR1 with ONP. The result was shown in Figure 6(D). The β-position carbon atom of the lactone ring carbonyl in the ONP can covalently bond well with the Sec at position 498 in the protein. This also proved once again that the interaction sites between ONP and pure TrxR were the β-position carbon atom on the lactone ring and the Sec at position 498 of the enzyme. In short, our data fully confirms that ONP with α, β-unsaturated ketone structure can well capture Sec residues to heavily inhibit TrxR activity, which provides strong evidence for explaining the above-mentioned essential intracellular processes.

### 3.7. Contribution of targeting TrxR to ONP cytotoxicity

**Figure 7.**
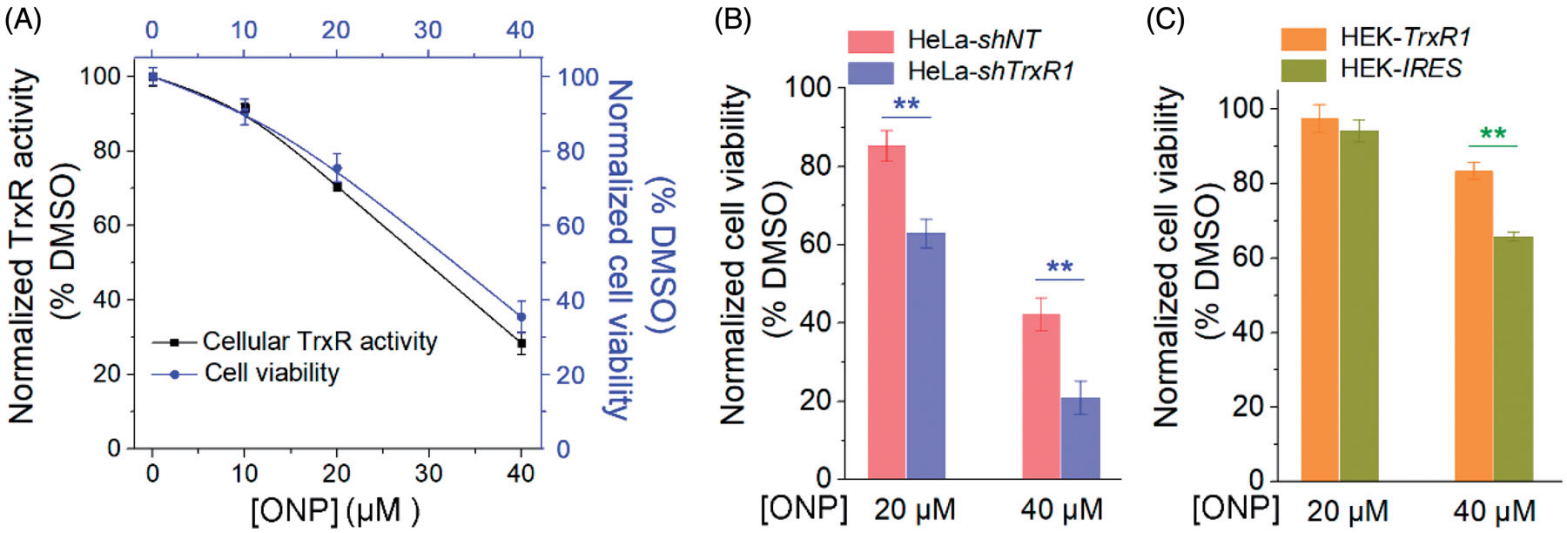
Contribution of targeting TrxR to ONP cytotoxicity. (A) The positive correlation between inhibition of HeLa cell viability by ONP and inhibition of intracellular TrxR activity by ONP. Different concentrations of ONP were incubated with HeLa cells for 24 h, and the intracellular TrxR activity was detected by the Trx-mediated endpoint insulin reduction assay. The HeLa cell survival rate under the same administration conditions was detected by the trypan blue exclusion staining method. (B) The difference in cytotoxicity of ONP towards the HeLa cell line knocked down TrxR1. ONP (20 and 40 μM) acted on HeLa-*shNT* and HeLa-*shTrxR1* cells for 24 h, respectively, and the cell viability was detected by the trypan blue exclusion staining method (average of three independent experiments). (C) The difference in cytotoxicity of ONP to HEK 293T cell line overexpressing TrxR1. The survival rate of HEK-*IRES* and HEK-*TrxR1* cells under the same experimental conditions as (B) was detected by the trypan blue exclusion staining method (average of three independent experiments). Data are expressed as mean ± S. E. of three experiments. **p < 0.01 versus the control groups.

Since we have well verified the interaction site of ONP and TrxR, and this interaction highly caused ONP to suppress both pure TrxR and intracellular TrxR activity. In particular, ONP inhibited TrxR activity in cells, inevitably limiting the function of cellular TrxR. Therefore, when the tumor cells were further stressed, the cellular anti-oxidation ability was partially weakened. As a consequence, the upper limit of tumor cell vulnerability to ROS toxicity may be broken, finally leading to oxidative stress-mediated carcinoma cell apoptosis. The biological behavior of ONP targeting TrxR reasonably contribute to the cytotoxicity of ONP towards tumor cells. As shown in Figure 7(A), the survival rate of ONP on HeLa cells we determined previously had a certain extent correlation with its inhibitory rate on intracellular TrxR activity. To validate this correlation, we employed the HeLa-*shTrxR1* that had previously knocked down TrxR expression in HeLa cells and the control cell line HeLa-*shNT* generated by transfecting non-targeted shRNA plasmids^54^. After ONP at a concentration of 20 and 40 µM acted respectively on HeLa-*shTrxR1* with low TrxR expression and HeLa-*shNT* with empty plasmid cells, the results showed that the cytotoxicity of ONP at the same concentration to HeLa-*shTrxR1* cells was remarkably increased compared with HeLa-*shNT* cells (Figure 7(B)), which means TrxR participated in the physiological process of ONP inhibiting the growth of HeLa cells. Correspondingly, overexpression of the functional TrxR protein in HEK-*TrxR1* cells rendered the cytotoxicity of ONP to decrease when ONP at a concentration of 40 µM was used to act on the HEK-*TrxR1* overexpressing TrxR and the control cell line HEK-*IRES* (Figure 7(C)). Attenuation of cytotoxicity by ONP towards the HEK-*TrxR1* cell line illustrated further that TrxR was involved in the intracellular process of ONP. Taken together, our data supported that targeting TrxR strongly contributes to the cytotoxicity of ONP.

### 3.8. Significance of ONP as a new TrxR inhibitor with anticancer activity

Isolation and identification of bioactive molecules in natural products and uncovering their action mechanisms are of great significance to the discovery and development of medicinal value for secondary metabolites and natural plants. As the first major family of dicots, *Asteraceae* contains numerous genera and species, and most of their medicinal value and secondary metabolites have been systematically studied^55^. However, as a new genus of *Asteraceae* discovered in recent years^3^, the secondary metabolites from *Shangwua* and their bioactivities have not been reported yet. Since *Shangwua* grows in a high-altitude hypoxic environment^3^, research on its secondary metabolites and medicinal value would inevitably receive attention.

**Figure 8.**
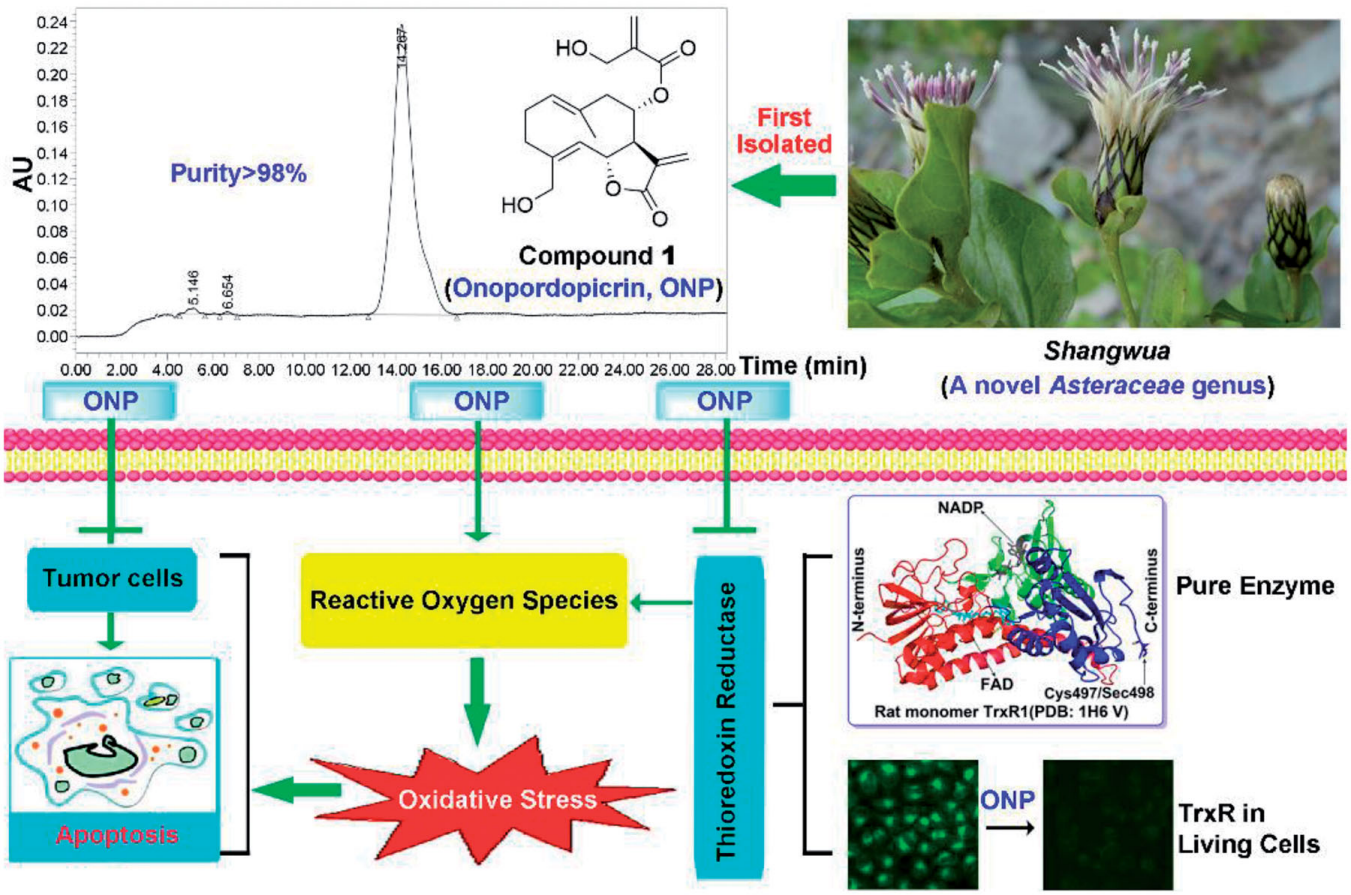
Revealing the mechanism of ONP isolated from the new genus *Shangwua* for targeting TrxR to induce oxidative stress-mediated apoptosis.

We herein isolated ONP, C**2** and C**3** from *Shangwua* for the first time, indicating that sesquiterpene lactones may be one of its primary secondary components (Figure 1). Intriguingly, these three compounds, *e.g.* ONP with significant cytotoxicity, C**2** with weaker cytotoxicity, and C**3** with almost no cytotoxicity, harbor significant differences in cytotoxicity towards tumor cells (Figure 2(A)). We revealed the reasons for the difference in their antitumor activity from the perspective of compound functional groups and described the antitumor mechanism of ONP targeting TrxR (Figures 5 and 6). Mechanistically, our results revealed that ONP selectively acts on the alteration of redox homeostasis of carcinoma cells, thereby selectively killing tumor cells *via* apoptosis (Figures 3 and 4). The ability of ONP to vary the redox homeostasis of carcinoma cells was connected with targeting intracellular main antioxidant enzyme TrxR (Figure 8). Importantly, we not only demonstrated the interaction site of ONP and TrxR but also confirmed that TrxR is involved in the cytotoxicity of ONP. Our data supported that α, β unsaturated ketone in the ONP and the Sec residues with stronger nucleophilicity of the enzyme are the sites of action of both. Selective inhibition of TrxR activity impaired TrxR function in cells, resulting in a series of changes in intracellular redox levels to contribute to the antitumor activity of ONP.

We first studied the secondary metabolites of the new genus *Shangwua* and discovered a TrxR inhibitor with antitumor activity from it. Our results provided a basis not only for the development of bioactive components of the genus *Shangwua* but also for the establishment of the medicinal value of the *Shangwua*. For the first time, we revealed the antitumor activity of the bioactive ingredient ONP and its antitumor mechanism targeting TrxR, inspiring the development of new pharmacological activities of ONP and providing support for the contribution of the target TrxR to the antitumor effect. Therefore, the discovery of ONP from *Shangwua* and the report of its antitumor activity are of great significance to the establishment of new genus resource components and medicinal value.

## 4. Conclusions

We isolated and characterized compounds ONP, C**2**, and C**3** from a new genus *Shangwua*, and evaluated the pharmacological function of ONP with interfering with cellular redox signaling. Further experiments demonstrated that ONP robustly inhibits TrxR, causing perturbation of cellular redox homeostasis with the favor of oxidative stress. Knockdown of the TrxR expression sensitizes cells to the ONP treatment while overexpression of the enzyme reduces the potency of ONP, underpinning the correlation of TrxR inhibition to the observed cytotoxicity of ONP. Our results for the first time revealed the bioactive ingredients and antitumor activity from the new genus *Shangwua* and served as the discovery of the secondary metabolites and the validation of medicinal value for *Shangwua*.
